# Management of Atrio-Esophageal Fistula Following Left Atrial Ablation

**DOI:** 10.14740/cr454e

**Published:** 2016-02-20

**Authors:** Tariq Yousuf, Hesam Keshmiri, Zachary Bulwa, Jason Kramer, Hafiz Muhammad Sharjeel Arshad, Rasha Issa, Daniel Woznicka, Paul Gordon, Pierre Abi-Mansour

**Affiliations:** a Department of Internal Medicine, Advocate Christ Medical Center, 105 Covington Ct, Oak Brook, IL 60523, USA; b Rosalind Franklin University of Medicine and Science, North Chicago, IL, USA; c Department of Cardiovascular Surgery, Advocate Christ Medical Center, Oak Lawn, IL, USA; d Department of Cardiology, Advocate Christ Medical Center, Oak Lawn, IL, USA

**Keywords:** Atrial ablation, Atrial fibrillation, Arrhythmia, Atrio-esophageal fistula

## Abstract

Currently, no guidelines have been established for the treatment of atrio-esophageal fistula (AEF) secondary to left atrial ablation therapy. After comprehensive literature review, we aim to make suggestions on the management of this complex complication and also present a case series. We performed a review of the existing literature on AEF in the setting of atrial ablation. Using keywords atrial fibrillation, atrial ablation, fistula formation, atrio-esophageal fistula, complications, interventions, and prognosis, a search was made using the medical databases PUBMED and MEDLINE for reports in English from 2000 to April 2015. A statistical analysis was performed to compare the three different intervention arms: medical management, stent placement and surgical intervention. The results of our systematic review confirm the high mortality rate associated with AEF following left atrial ablation and the necessity to diagnose atrio-esophageal injury in a timely manner. The mortality rates of this complication are 96% with medical management alone, 100% with stent placement, and 33 % with surgical intervention. Atrio-esophageal injury and subsequent AEF is an infrequent but potentially fatal complication of atrial ablation. Early, prompt, and definitive surgical intervention is the treatment of choice.

## Introduction

Treatment of atrial fibrillation (AF) consists of heart rate control, rhythm control, and anticoagulation. Left atrial catheter ablation with either cryotherapy or radiofrequency is now accepted as a treatment for symptomatic drug-refractory AF. With an increase in left atrial ablation procedures, there has also been a concomitant rise in post-procedural complications.

We present a review of the previous literature that demonstrates the dire complication of atrio-esophageal fistula (AEF), which is defined as an abnormal connection between the esophagus and atrium of the heart following left atrial ablation.

## Case Reports

### Case 1

A 77-year-old female with a past medical history of paroxysmal AF of 8 years duration presented to the hospital with worsening symptoms of palpitations and shortness of breath over the past 6 months. Over the previous 3 months, she had uncontrolled symptomatic arrhythmias despite being on dronedarone, which was then switched to amiodarone. After the switch, she suffered from additional symptoms including fatigue, constipation, loss of appetite, and dry cough. She underwent left atrial cryotherapy ablation for treatment of AF. On post-operative day 1, she complained of dysphagia, which resolved using empiric medical therapy with pantoprazole and sucralfate.

**Figure 1:**
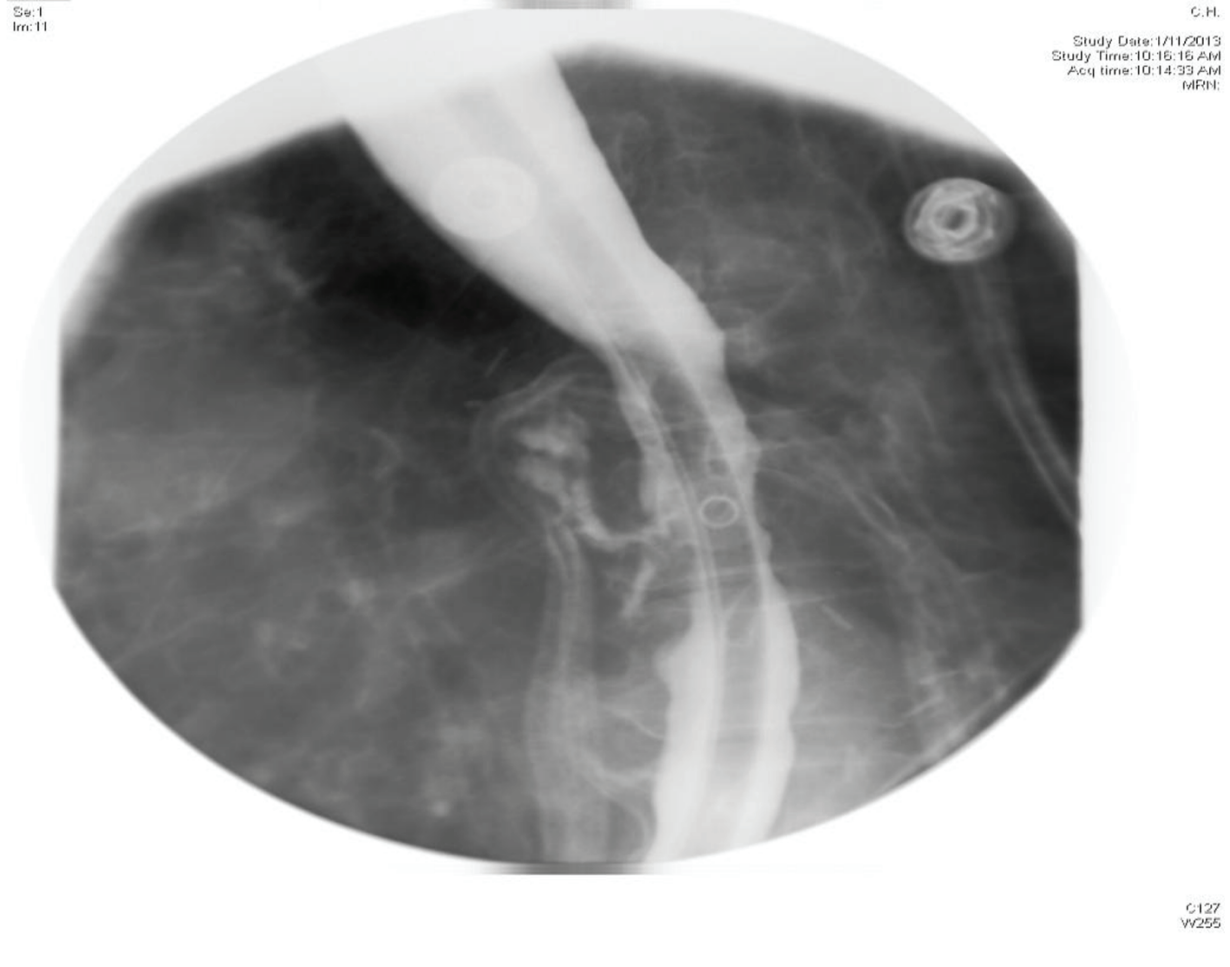
X-ray esophagus with gastrografin showing sinus tracts extending from anterior aspect of esophagus.

Twenty days after the ablation, the patient was re-admitted with fever, chills, and difficulty swallowing. An esophagram with gastrografin demonstrated a contrast leak anterior to the esophagus at the level of the left atrium but without an obvious connection (Fig. 1). She developed new-onset weakness in her left extremities. Computed tomography angiography (CTA) revealed multifocal emboli. A transthoracic echocardiogram (TTE) did not show any evidence of a clot in the atrium. With a high suspicion for an AEF, a thoracic CT was performed and confirmed a fistulous tract between the esophagus and left atrium.

**Figure 2:**
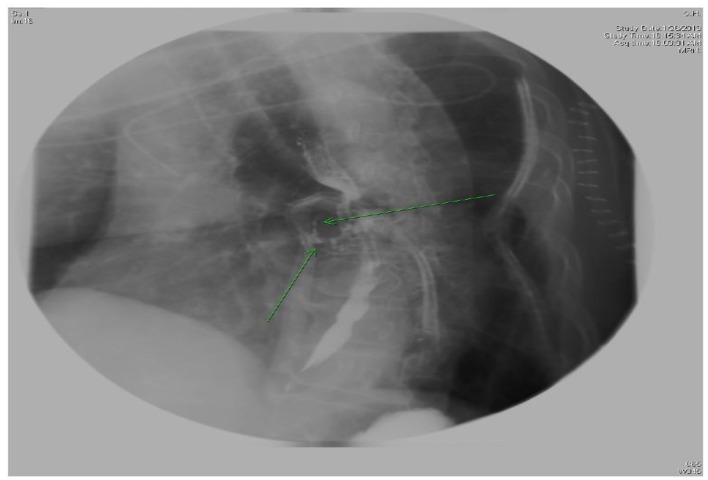
X-ray esophagus with gastrografin showing two sinus tracts extending from anterior aspect of esophagus at level T7.

A left posterior lateral thoracotomy was performed for surgical repair of the existing fistula. Post-operatively, repeat esophagram with gastrografin was ordered and demonstrated additional sinus tracts extending from the thoracic segment of the esophagus to the left atrium (Fig. 2). Due to the persistent fistula, the patient underwent another surgery. The patient was not sent home on oral anticoagulation therapy. Two weeks following the second surgery, the patient had several bouts of intractable coughing. An esophagram after this episode re-demonstrated a fistulous tract. Despite heroic measures, the patient’s condition deteriorated and she ultimately expired 3 days later secondary to septic shock.

### Case 2

**Figure 3:**
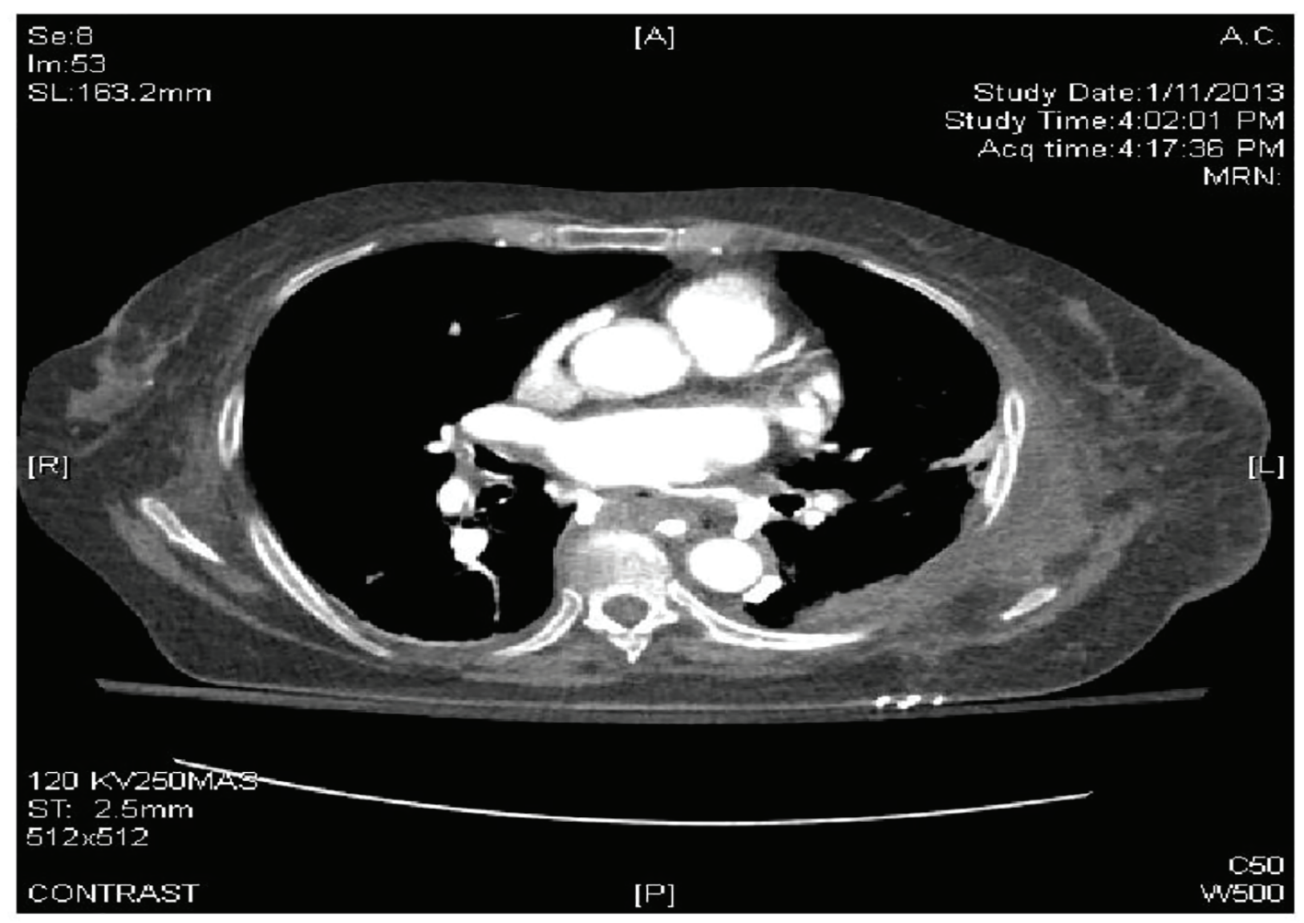
CTA of the chest and abdomen identified esophageal perforation with a fistulous tract to the left atrium.

A 64-year-old male underwent cryoablation of all four pulmonary veins for symptomatic drug refractory AF and was discharged home the same day on rivaroxaban and amiodarone for 3 months as well as an event monitor for 3 weeks. Later that night, the patient developed pleuritic chest pain. He was found to be in atrial flutter in the emergency department. A CTA of the chest and abdomen identified esophageal perforation with a fistulous tract to the left atrium (Fig. 3). Echocardiogram at this time showed a moderate to large pericardial effusion with no evidence of hemodynamic compromise.

**Figure 4:**
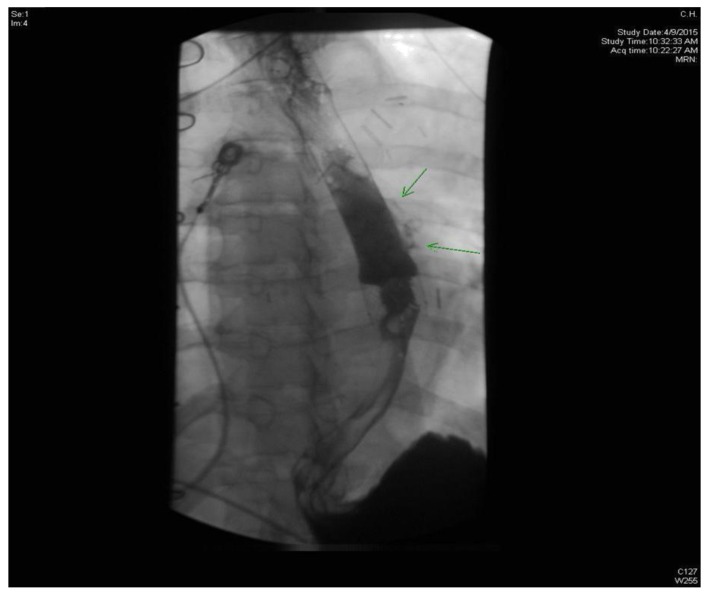
Esophagogram showing extravasation of contrast from posterolateral aspect of esophagus.

Median sternotomy was performed 5 days post-ablation in order to drain the pericardial effusion of purulent fluid. An esophageal stent was also placed for the treatment of an AEF. Pericardial fluid cultures grew group A *Streptococcus* and the patient was started on antibiotics. Although he improved clinically, an esophagram revealed a persistent AEF (Fig. 4). The patient was sent to a rehabilitation facility.

The patient was hospitalized and found to have intermittent febrile episodes with hypotension and three transient episodes of left-sided weakness. The patient was in AF with rapid response; therefore, he was maintained on amiodarone and a heparin drip.

**Figure 5:**
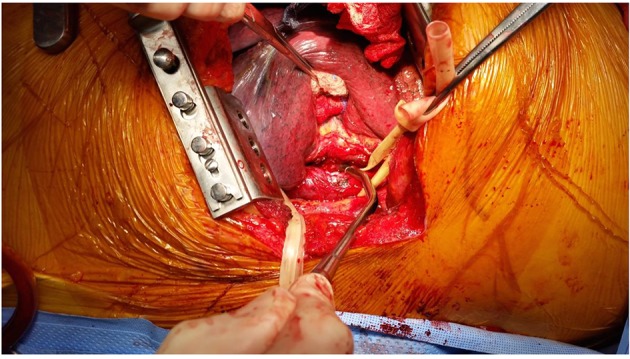
Two-centimeter defect in the esophagus.

**Figure 6:**
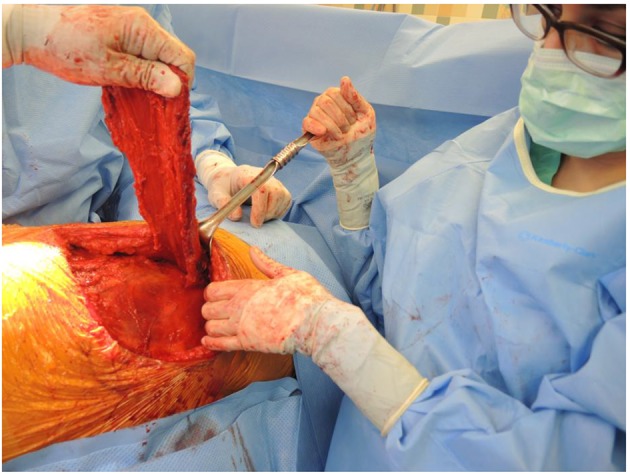
Esophagus wrapped circumferentially with latissimus flap.

A TTE demonstrated a mobile echogenic structure within the left atrium consistent with a thrombus. He underwent sternotomy with removal of the left atrial thrombus and closure of a patent foramen ovale. The patient was transferred to our facility to undergo repair of the newly demonstrated AEF. Figure 5 demonstrates the 2-cm defect in the esophagus (Fig. 5). It was accomplished with surgery consisting of an autologous pericardial patch with suture repair of the esophageal defect and latissimus dorsi muscle interposition flap placement (Fig. 6). The esophagus was circumferentially wrapped with the latissimus flap. The esophageal stent was also removed. He tolerated the surgery well. Heparin drip was started immediately post-operatively. The patient was discharged to a rehabilitation facility. Patient was followed up several months afterwards and has continued to do well post-operatively.

### Discussion

The indications and application of left atrial ablation in the treatment of AF have progressively expanded in the past decade [1, 2]. A single ablation procedure was successful in 50% of patients and additional ablation procedures increased long-term freedom from AF to 80% [3]. Nevertheless, complication rates following ablation have mounted with rates varying amongst studies from 0.8% to 6% [1, 4]. When patients consent to catheter ablation, they must understand the risks associated with the procedure. Major complications include cardiac tamponade (1.31%), thromboembolic events (stroke 0.23%, transient ischemic attacks 0.71%), pulmonary vein stenosis (0.29% requiring dilation), esophageal injury (AEF 0.04%), and death (0.15%) [1, 4]. Esophageal injury can vary greatly from one patient to the next. The spectrum of injury ranges from esophageal erythema, ulcer-like changes in the esophageal wall, peri-esophageal nerve injury, esophageal perforation, and the development of an AEF.

Although AEF is a rare complication, it carries a higher rate of morbidity and mortality compared to all other complications related to AF catheter ablation [1]. Due to its anatomic location relative to the posterior left atrium (0.9 ± 0.2 mm) [4], the anterior esophagus is particularly prone to surgical injury [5]. Esophageal complications have been reported in up to 47% of patients who underwent catheter ablation [4]. The most notorious and fatal of these esophageal complications is AEF. Table 1 [6-48] provides a comprehensive summary of all documented cases of AEF (Table 1).

**Table 1:** Descriptions of Published Case Reports and Case Series With AEF After Ablation for Atrial Fibrillation

Lead author, year	NP	Age	Sex	LAOC	Clinical manifestations	Treatment	Outcome
Mohr, 2002 [6]	3	NR	NR	3 - 6	Neurological symptoms (2): stroke (2); GI bleeding	Surgery (2)	Survived (2) (surgery); died
Kottkamp, 2002 [7]	1	NR	NR	10	Fever (septicemic), neurological symptoms	Surgery	Survived
Doll, 2003 [8]	4	36 - 62	M (3), F (1)	3 - 12	Fever (2), hematemesis, chest pain, neurological symptoms (2): TIA	Surgery (3), antibiotics, died during gastroscopy	Survived (3), died
Sonmez, 2003 [9]	1	58	F	22	Fever, shivering, neurological symptoms: numbness in right arm	Surgery	Died
Pappone, 2004 [10]	2	36, 59	M (2)	3, 2	Fever (2), rigors, chest pain (2), neurological symptoms (2): convulsions, loss of consciousness, left-sided hemiparesis, weakness, grand mal seizure, stroke	Surgery, antibiotics	Survived; died
Scanavacca, 2004 [11]	1	72	M	20	Fever, hematemesis, neurologic symptoms: loss of consciousness, tonic-clonic seizures; dysphagia, anorexia	Antibiotics	Died following endoscopy
Dagres, 2006 [12]	5	35 - 76 (mean 51)	M (4), F (1)	8 - 28	Fever (3), chest pain (2), neurological symptoms (3): hemiparesis (3), grand mal seizure, aphasia	Surgery (3), died before surgery, died during gastroscopy	Survived (3) (surgery), died (2)
Schley, 2006 [13]	1	37	M	25	Fever, neurological symptoms: grand mal seizure, status epilepticus, loss of consciousness	Died before surgery	Died
Cummings, 2006 [14]	9	NR	M (4), F (5)	10 - 16 (mean 12.3)	Fever (9) (sepsis), chest pain (2), neurological symptoms (8), GI bleeding (3), occult bleeding (5)	Surgery (3), 1 died before surgery	Died (9)
Gerstenfeld, 2007 [15]	1	NR	NR	21	NR	NR	Died
Malamis, 2007 [16]	1	59	M	35 (5 weeks)	Fever (sepsis), neurological symptoms: confusion, altered mental status	Surgery (died in surgery)	Died
Preis, 2007 [17]	1	56	M	38	Fever, chills, neurological symptoms: right arm weakness, aphasia, stroke	Surgery	Survived
Dixit, 2008 [18]	1	NR	F	14	Fever, hematemesis, neurological symptoms: coma; nausea	Endoscopy	Died
Borchert, 2008 [19]^a^	1	59	M	10	Fever (sepsis), chest pain, neurological symptoms: seizure, coma	Surgery	Died
Ouchikhe, 2008 [20]	1	58	M	21 (3 weeks)	Fever (septic shock), neurological symptoms: meningitis, confusion; vomiting	Antibiotics	Died
Hazell, 2009 [21]	1	72	M	24	Rigors, neurological symptoms: weakness, collapse, loss of consciousness, disorientation	Stent placement, antibiotics	Died
Cappato, 2009 [22]	7	NR	NR	NR	NR	NR	Survived (2); died (5)
Ghia, 2009 [23]	6	NR	NR	NR	Neurological symptoms (6): cerebrovascular events (6)	NR	Survived (1); died (5)
Khandhar, 2010 [24]	1	46	M	27	Fever, neurological symptoms: transient left-sided hemiparesis, aphasia	Surgery	Survived
Gilcrease, 2010 [25]	1	61	M	10	Fever, hematemesis, chest pain, neurological symptoms: altered and variable mental status, seizures; fatigue, dysphagia	Surgery	Died
Baker, 2010 [26]	1	65	F	20	Fever, neurological symptoms: confusion, tonic-clonic seizure, stroke; GI bleeding, MI	Died before surgery	Died
Cazavet, 2010 [27]	1	35	M	38	Fever, chest pain, neurological symptoms: left-sided hemiplegia, convulsive crisis; vomiting	Stent placed and migrated requiring surgical intervention, antibiotics	Survived
Neven, 2010 [28]^a^	1	69	M	31	Hematemesis, neurological symptoms: seizures; pneumonia	None	Died
Siegel, 2010 [29]	1	46	M	3	Fever (sepsis), neurological symptoms: right-sided hemiparesis, rigors, near-syncope, myoclonic jerking, confusion, positive Babinski signs	Surgery, antibiotics	Survived
Zellerhoff, 2011 [30]	1	63	M	14 (2 weeks)	Fever, neurological symptoms: muscles weakness in arms and legs, left-sided hemiparesis	Stent placement	Died
St Julien, 2011 [31]	1	59	M	42 (6 weeks)	Fever, chest pain, neurological symptoms: headache, altered mental status, aphasia, right hemiplegia, TIA, seizures; GI bleeding, diaphoresis	Surgery, antibiotics	Survived
Tancevski, 2012 [32]	1	45	M	42 (6 weeks)	Fever, neurological symptoms: weakness, sensory loss of the right limbs	Surgery, antibiotics	Survived
Haggerty, 2012 [33]	1	27	M	22	Fever, chills, hematemesis	Surgery, antibiotics	Survived
Hartman, 2012 [34]	1	62	M	30 days (1 month)	Fever, chills, neurological symptoms: rigors, syncope, left-sided hemiplegia, grand mal seizure; odynophagia, nausea, vomiting, abdominal pain	Surgery, antibiotics	Survived
Stockigt, 2012 [35]	1	78	M	28 days (4 weeks)	Fever, shivering, neurological symptoms: stroke, persistent vegetative state; coughing	Surgery, antibiotics	Survived
Tan, 2013 [36]	1	67	F	20	Fever, neurological symptoms: numbness in left foot, unresponsiveness; nausea	Antibiotics	Died
Rivera, 2013 [37]	1	50	F	28 (4 weeks)	Hematemesis	Surgery	Survived
Aryana, 2013 [38]	1	55	F	21 (3 weeks)	Neurological symptoms: seizures, left hemiparesis	Surgery	Died
Shim, 2013 [39]	1	68	M	50	Fever (sepsis), neurological symptoms: stroke	None	Died
Mohanty, 2014 [40]	9	46 - 62 (mean 53.3)	M (8), F (1)	14 - 42 (2 - 6 weeks) (mean 27.2)	Fever (7) (sepsis (3)), rigors (2), chest pain (7), neurological symptoms (9): stroke/TIA (9), confusion, weakness (3), seizure (2), hemiparesis (3), blindness (2), convulsions, altered mental status; GI bleeding (2), dysphagia	Stent placement (5), surgery (4)	Died (5) (stent placement); survived (4) (surgery)
Rajapaksha, 2014 [41]	1	43	M	NR	Chest pain, hematemesis, hemoptysis, neurological symptoms: right arm weakness; throat pain, dyspnea, palpitations, nausea	None	Died
Vilades Medel, 2014 [42]	1	31	M	28 (4 weeks)	Fever (septic shock), hemoptysis, neurological symptoms: headache, absence seizures, intracranial bleed	Surgery	Died
Lim, 2014 [43]	1	61	M	30 (1 month)	Fever, chills, rigors	Stent placement	Died
Shiraishi, 2014 [44]	1	49	M	31	Fever, neurological symptoms: seizures	Endoscopic snaring, antibiotics	Survived
Kawasaki, 2014 [45]	3	58, 48, 75	F, M, F	12, 3, 3	Fever (2), chills (2), rigors (3), neurological symptoms (3): TIA, collapse, left-sided hemiplegia/paresis, weakness in right arm, facial drooping with slurred speech, altered mental status; dysphagia, fatigue, palpitations, abdominal pain	Surgery (2), antibiotics (3)	Died (3)
Schouver, 2014 [46]	1	63	M	15	Fever (septic shock), neurological symptoms: stroke; ST elevation inferior coronary syndrome, acute renal failure, DIC	NR	NR
Koa-Wing, 2014 [47]	1	45	M	21 (3 weeks)	Neurological symptoms: unconsciousness, stroke	None	Died
Hirji, 2015 [48]	1	46	M	7 (1 week)	Fever, neurological symptoms: confusion, right-sided weakness, difficulty walking, meningitis	Surgery, antibiotics, postoperative hyperbaric oxygen therapy (HBOT)	Survived

NP: number of patients; M: male; F: female; PR: procedure: LAOC: latency between ablation and onset of clinical manifestations (days); 1 week = 7 days; 1 month = 30 days; NR: not reported. ^a^Borchert, 2008 and Neven, 2010 used HIFU (high-intensity focused US) to treat atrial fibrillation.

Prompt diagnosis of AEF is paramount as emergent intervention is required [49]. The clinical presentation of AEF includes but is not limited to fever, chest pain, dysphagia, melena, hematemesis, and sepsis. AEF usually presents between 3 days and 5 weeks post-ablation [50]. Many patients with AEF present with neurological manifestations including stroke, TIA, seizures, and meningitis [49, 50]. The AEF can act as a single-way valve for air and emboli leading to neurologic manifestations [50]. Diagnosis requires high clinical suspicion for AEF and knowledge of the complications of catheter ablation. If AEF is suspected, water-soluble contrast esophagram may illustrate a fistulous tract. In addition, X-ray or CT of the chest may aid in identifying pneumopericardium and pneumomediastinum.

Relative contraindications exist for upper endoscopy and TEE in order to limit food and air embolism, enlargement of the fistula, and further esophageal damage [50, 51]. Furthermore, oral intake should be avoided to prevent postprandial food embolism. In order to prevent AEF formation, several protective measures have been suggested. These include gastric acid suppression, esophageal temperature monitoring, mechanical deflection of the esophagus, and thermal insulation of the esophagus [10].

Although there are no current guideline recommendations about treatment with stents or surgical fistula takedown [2], our review indicates a dramatic response to definitive surgical intervention, which shows a decreased mortality rate from 100% to 32.4% (Table 2).

**Table 2:** Summary Table

Total number of patients with reported AEF	82
Males	44
Females	16
Not specified	22
Average reported age	54.0 years old
Average latency between therapeutic procedure and onset of clinical manifestations *Borchert, 2008 and Neven, 2010 used HIFU (high-intensity focused US) to treat atrial fibrillation	20.1 days
Clinical manifestations	74 patients’ manifestations reported
Neurological symptoms Stroke/TIA Confusion Seizure Paresis Sensory disturbance	65 (87.8%)
Fever/chills/shivering/rigors	54 (73.0%)
Chest pain	19 (25.7%)
Hematemesis/hemoptysis	9 (12.2%)
GI bleeding	8 (10.8%)
Therapeutic intervention of AEF	
Stent placement	8
Surgery	36
Medical management alone	23
Mortality rate following intervention	
Stent placement	100% (8/8)
Surgery	33.3% (12/36)
Medical management	95.6 % (22/23)
Total reported mortalities	53
Total reported survivors	29

In a non-randomized control trial, stenting alone was initially chosen because it is less invasive [5]. Due to poor outcomes, the remaining patients were treated with surgical intervention, which showed a significant improvement in mortality. From review of the literature, patients who received stent placement alone had significantly higher mortality because stents cannot prevent embolic events with an established AEF [52]. Ellis et al demonstrated stenting as a preventative measure post-esophageal injury without AEF [10].

The question will arise regarding the unstable patient with AEF. Conventional standards suggest that surgery may be associated with higher risks. However, our review of the literature shows that most patients with AEF who received stent placement or medical therapy alone without surgical intervention will die. Therefore, we believe that even the unstable patient should be taken to the operating room in a timely manner. Stenting may be used as a temporary bridge to surgical intervention but is by no means a definitive therapy. Maintenance of anticoagulation is recommended due to high risk of left atrial thrombus formation and high incidence of cardio-embolic events. Early initiation of anticoagulation after surgical repair may decrease the incidence of post-repair stroke.

A multicenter prospective study may be warranted to further validate our conclusions. This would help elucidate any biases of patient population, physician preference, and operator proficiency.

### Conclusion

Esophageal injury and subsequent fistula formation are among the most frequent and fatal complications of left atrial ablation therapy for symptomatic AF. Screening for patients at increased risk as well as peri-procedural attempts at minimizing the risk of esophageal injury needs to be pursued. Ultimately, survival after post-ablative esophageal injury is likely correlated to high diagnostic suspicion for such injury, prompt diagnosis, and urgent surgical intervention rather than stent placement or medical management for this dreaded complication.
